# A qualitative study on the delayed manifestation of night shift-related intrusive memories in non-shift nurses with a history of night shift work

**DOI:** 10.3389/fpubh.2026.1938159

**Published:** 2026-09-17

**Authors:** Xiaona Gai, Zhaona Hao, Chuanli Jiang, Meng Liu, Qing Yang, Hongmei Xu

**Affiliations:** 1 School of Nursing, Shandong Medical and Pharmaceutical University, Yantai, Shandong, China; 2 Outpatient Department, Yantai Affiliated Hospital of Shandong Medical and Pharmaceutical University, Yantai, Shandong, China; 3 Department of Neurology II, Yantai Affiliated Hospital of Shandong Medical and Pharmaceutical University, Yantai, Shandong, China; 4 Department of Otolaryngology, Yantai Affiliated Hospital of Shandong Medical and Pharmaceutical University, Yantai, Shandong, China; 5 Office of Science and Technology, Shandong Medical and Pharmaceutical University, Yantai, Shandong, China

**Keywords:** intrusive memories, night shifts, nurses, occupational health, phenomenological research, qualitative research

## Abstract

**Introduction:**

The adverse effects of night shift work on the physical and mental health of nurses have been widely documented. However, little is known about whether these psychological experiences persist after nurses cease working night shifts or whether some of these experiences subsequently manifest as intrusive memories.

**Objective:**

To explore the characteristics of the delayed manifestation of night shift-related intrusive memories and the associated experiences among non-shift nurses with a history of night shift work.

**Methods:**

A descriptive qualitative study design was employed using a descriptive phenomenological approach. Fifteen non-shift nurses with a history of night shift work who were currently working fixed day shifts at a Grade III Class A hospital in Shandong Province were recruited for semi-structured interviews. Colaizzi’s seven-step method was used for data analysis. The findings were reported in accordance with the Consolidated Criteria for Reporting Qualitative Research (COREQ) guideline.

**Results:**

Among nurses who reported night shift-related intrusive experiences after the cessation of night shift duties, these experiences may persist for months or even years and may be reactivated by specific situational cues, accompanied by emotional responses. Four themes were identified: Night Shift-Related Psychosocial Stressors; Characteristics of Night Shift-Related Intrusive Memories; Contextual Cues and Persistent Experiences Following Night Shift Work; and Coping and Meaning Reconstruction After Night Shift Experiences.

**Conclusion:**

Among nurses who reported night shift-related intrusive experiences after the cessation of night shift duties, these experiences were characterized by delayed onset, involuntary recurrence, and situational dependence. These findings highlight the importance of recognizing the psychological experiences of nurses with a history of night shift work and suggest the need for appropriate occupational mental health support and adaptation strategies.

## 1 Introduction

Night shifts are an integral component of nursing work schedules and are essential for ensuring the continuous provision of healthcare services on a 24-h basis (1). Previous studies have demonstrated that nurses engaged in long-term night shift or rotating shift work are more susceptible to psychological disorders, including sleep disturbances, chronic fatigue, anxiety, and depression. Occupational burnout and reduced quality of life are also common among this population (2). Owing to the continuous nature of healthcare delivery, nurses are among the occupational groups exposed to night shift work (3, 4). Existing research has primarily focused on nurses who are currently engaged in night shift or rotating shift work, with most studies examining the effects on physiological burden, psychological stress, and career development (5). Evidence has shown that night shift work not only disrupts circadian rhythms but may also have long-term adverse effects on sleep health and broader occupational health outcomes (6, 7). From the perspective of occupational health, exposure to night shift work represents an important occupational health concern for nurses. Its impact extends beyond individual health to influence nursing care quality and patient outcomes (8).

As career trajectories, job responsibilities, and personal circumstances change, some nurses gradually transition from night shift work to relatively fixed daytime work schedules. Compared with continued rotating shift work, transitioning to daytime schedules may reduce some of the adverse effects associated with shift work (9). Nevertheless, previous studies among healthcare workers have suggested that stressful occupational experiences may be re-experienced through involuntary intrusive memories and emotional responses when individuals encounter reminders associated with previous events (10). For example, specific environmental cues or reminders associated with previous night shift experiences may act as triggers, causing individuals to re-experience related psychological responses. These experiences are often accompanied by increased tension, anxiety, heightened alertness, sleep disturbances, or avoidance behaviors (11–13). These observations suggest that the psychological effects of stressful occupational experiences do not necessarily disappear after the original exposure has ended. Instead, they may persist as relatively stable memory traces that can be reactivated under specific contextual conditions (14).

One plausible explanation for this phenomenon is that negative workplace experiences are encoded within the memory system and subsequently reactivated by specific situational cues. Intrusive memories generally refer to fragments of distressing experiences that recur involuntarily and repeatedly in an individual’s consciousness and are often accompanied by marked emotional changes and physiological responses (15). Previous studies have shown that intrusive memories are not confined to individuals who have experienced conventional traumatic events. People working in high-stress occupations or exposed to persistent occupational stress may also develop stable representations of negative memories that can be reactivated under appropriate circumstances (16). Exposure to external stimuli resembling the original situation may reactivate previously suppressed memories, thereby eliciting corresponding psychological and physiological responses (17). Furthermore, studies have indicated that even long after stressful events have ended, healthcare professionals, emergency service personnel, and other workers in high-stress occupations may continue to experience work-related psychological effects that can be reactivated under specific environmental conditions (18). Long-term exposure to night shift work, sustained workload, nocturnal working schedules, and related occupational stressors may contribute to persistent negative psychological experiences and intrusive memories among healthcare workers. Consequently, even after they have ceased working night shifts, exposure to cues associated with previous stressful occupational experiences may reactivate these intrusive memories (19).

Despite the substantial body of evidence regarding the adverse health effects of night shift work, most previous studies have focused on its immediate consequences, including sleep disturbances, anxiety, depression, occupational burnout, and overall deterioration in health (20–22). In recent years, research on the long-term psychological consequences among nurses has gradually expanded to include the effects of occupational stress on mental health, the development of profession-related risk factors, and occupational well-being (23). Nevertheless, limited attention has been paid to nurses who have discontinued night shift work, particularly regarding their long-term experiences and developmental trajectories, and whether the psychological effects of night shift work persist in the form of stable mental representations. Furthermore, little is known about why intrusive memories related to night shift work continue to occur after nurses have stopped working night shifts, the circumstances under which these memories are triggered, or how individuals respond to them. To date, few qualitative studies have investigated the delayed manifestation of night shift-related intrusive memories among non-shift nurses with a history of night shift work. Therefore, this study adopted a descriptive phenomenological approach to explore the delayed emergence of night shift-related intrusive memories among non-shift nurses with previous night shift experience. The findings may contribute to improving occupational health management models and the development of long-term psychological support and occupational adaptation strategies for this population.

## 2 Research methods

### 2.1 Research design

This study employed a descriptive phenomenological design. Data were collected through face-to-face semi-structured interviews to explore the experiences and meanings associated with the delayed manifestation of night shift-related intrusive memories among non-shift nurses with previous night shift experience. Descriptive phenomenology is grounded in the lived experiences of participants and focuses on their processes of understanding, perception, and meaning construction (24). This approach is appropriate for exploring the persistent influence of night shift experiences after nurses have ceased night shift work and for understanding the meanings attributed to these experiences (25, 26). This study specifically focused on the process through which nurses re-experienced memories, emotions, and physiological responses related to previous night shift work months or even years after discontinuing night shifts. It also explored how these experiences influenced their work, daily lives, and professional perspectives. The interview guide was developed according to the study objectives and based on a review of the relevant literature. Open-ended questions were used to encourage participants to describe their personal experiences, and follow-up questions were asked when necessary to obtain comprehensive and in-depth information throughout the interview process (27).

### 2.2 Study participants

The study was conducted at a Grade III Class A hospital in Shandong Province. To ensure diversity in age, years of professional experience, night shift experience, and job position, participants were recruited using purposive sampling combined with maximum variation sampling to obtain rich and diverse data (28). Eligible non-shift nurses with a history of night shift work were recruited with the assistance of the nursing administration department. Before participation, all eligible nurses were informed of the study objectives, interview procedures, and the contents of the informed consent form. All participants voluntarily agreed to participate and provided written informed consent.

The inclusion criteria were as follows: (1) currently employed on a fixed day shift schedule and not expected to undertake night shifts within the following 6 months; (2) previous experience of night shift work; (3) having discontinued night shift work for at least 3 months and having experienced night shift-related intrusive experiences after discontinuation of night shifts; and (4) ability to communicate clearly and describe their experiences and feelings. The exclusion criteria were as follows: (1) currently engaged in night shift or rotating shift work; (2) impaired cognitive or decision-making capacity due to mental or cognitive disorders; and (3) negative emotional experiences unrelated to previous night shift work.

Sample size was determined according to the principle of data saturation, and a total of 15 participants were ultimately included. All participants were currently working fixed day shifts and had previous experience of night shift work. They were recruited from outpatient departments and other clinical nursing departments and represented a wide range of night shift experience and varying durations since discontinuation of night shift work (Table 1).

**Table 1:** Presents the demographic characteristics of the participants.

Number	Age (years)	Years of nursing experience (years)	Years of night shift work	Years away from night shifts	Education	Current department
P1	36	15	8	7	Undergraduate	General Outpatient Clinic
P2	34	11	6	5	Undergraduate	Admission Preparation Center
P3	35	12	5	7	Undergraduate	Medical Services Appointment Center
P4	40	16	5	11	Undergraduate	Surgical Outpatient Clinic
P5	56	30	17	13	Undergraduate	Chronic Disease Clinic
P6	55	34	20	14	Undergraduate	Surgical Dressing Room
P7	33	14	7	7	Undergraduate	Ophthalmology Clinic
P8	50	31	18	13	Undergraduate	Internal Medicine Outpatient Clinic
P9	37	17	9	8	Undergraduate	Hemodialysis Unit
P10	33	10	3	7	Master’s Degree	Department of Otolaryngology
P11	32	11	7.5	3.5	Undergraduate	Central Sterile Supply Department
P12	54	34	17	17	Undergraduate	Health Screening Center
P13	31	13	10	3	Undergraduate	Gastroenterology Suite
P14	30	11	7	4	Undergraduate	Ultrasound Room
P15	44	20	15	5	Undergraduate	Thyroid and Breast Surgery Department

The time since participants had discontinued night shift work ranged from 3 to 17 years, with a median duration of 7 years.

### 2.3 Data collection

Face-to-face semi-structured interviews were conducted between February and April 2026. The interview schedule was arranged according to participants’ availability, and interviews were conducted during working hours. The interview location was selected by the participants; however, most interviews took place in quiet and private areas within the hospital to minimize external distractions. All participants provided written informed consent before participation. After receiving a full explanation of the study, all participants voluntarily agreed to have the entire interview audio-recorded.

To minimize potential power imbalances, the interviewer was not in a direct supervisory relationship with any participant. Although the nursing management department assisted with communication and coordination with relevant clinical departments, the decision to participate was entirely voluntary and the management personnel were not involved in participant selection, interviews, or access to interview data. Participants were informed that their participation or withdrawal would not affect their employment status, performance evaluation, or professional development. All interview data were kept confidential and used solely for research purposes.

The research team consisted of one chief nurse, one deputy director of the outpatient department, two ward head nurses, and one master’s student in nursing. The four clinical nursing staff members had extensive experience in nursing management and clinical practice, and all five research team members received training in qualitative research methods before the study commenced. The master’s student in nursing was responsible for conducting the semi-structured interviews, while the other team members participated in study design, data analysis, discussion of the findings, and quality control. Pilot interviews were conducted before the formal interviews with three nurses who met the inclusion criteria. Based on the findings of the pilot interviews, the interview guide was revised to produce the final version (Table 2). During the formal interviews, the interviewer used the interview guide while asking follow-up questions according to participants’ responses to obtain more comprehensive and detailed information. The duration of the interviews ranged from approximately 30 to 50 min. No individuals other than the interviewer and the participant were present during the interviews, and repeat interviews were not conducted.

**Table 2:** Interview guide.

Interview outline
1. Kindly describe your experience of working night shifts, and your overall impressions and what this experience has meant to you professionally and personally.
2. Since you stopped working night shifts, have you ever had experiences in which you found yourself thinking about situations related to night shifts? Please describe one representative situation and what happened during it.
3. What feelings, thoughts, and/or physical reactions tend to accompany these instances when they recur, and what do these reactions signify for you?
4. Please explain, in terms of certain situations, which environmental cues, personal factors, auditory stimuli or specific events are more likely to cause the experiences mentioned above.
5. How may these experiences have impacted your job performance, daily functioning, relationships, and view of your career?
6. In case of such experiences, how do you usually cope and adapt? What methods have worked best to you? What support would you expect the hospital or its management model to provide?

Audio recordings were transcribed verbatim within 24 h after each interview, and data collection and analysis were conducted concurrently. Data saturation was considered to have been achieved when no new themes, subthemes, or meaningful information emerged from three consecutive interviews. The decision regarding data saturation was made through discussions among the research team, including the interviewer and researchers involved in data analysis, after reviewing the interview transcripts and coding results. Participant recruitment was discontinued once consensus was reached (29).

### 2.4 Data analysis

The interview recordings were transcribed verbatim and checked for completeness and accuracy within 24 h after each interview. The transcripts were anonymized using numerical codes and imported into NVivo 15 software for data management and analysis. Data were analyzed using Colaizzi’s seven-step method (30). The researchers repeatedly reviewed the interview transcripts, identified significant statements, generated initial codes, and subsequently developed themes and subthemes based on conceptual relationships among the coded data. Two researchers independently performed the data analysis. Any discrepancies in data interpretation were resolved through discussion until consensus was reached. Ultimately, four themes and 12 subthemes describing the delayed emergence of intrusive memories among non-shift nurses with a history of night shift work were identified. An example of the analytical process based on Colaizzi’s seven-step phenomenological analysis, illustrating the progression from significant statements to formulated meanings, subthemes, and themes, is provided in Supplementary Table 1.

### 2.5 Research rigor

The study followed the quality criteria for qualitative research proposed by Shenton, including credibility, transferability, dependability, and confirmability (31). To enhance the credibility of the findings, the interview data were repeatedly reviewed throughout the research process. Regular discussions among the research team, peer review of the findings, and comprehensive documentation of all research materials were undertaken to ensure methodological rigor. To enhance transferability, detailed descriptions of the study setting, participant characteristics, sampling strategy, and research procedures were provided. The findings are reported in accordance with the Consolidated Criteria for Reporting Qualitative Research (COREQ) (32).

### 2.6 Ethical considerations

This study was approved by the Medical Ethics Committee of Yantai Affiliated Hospital of Shandong Medical and Pharmaceutical University (Approval No. LW-20260213012) and was conducted in accordance with the Declaration of Helsinki (33). All participants provided written informed consent before participation. The data were anonymized using coded identifiers and were used solely for academic research purposes. Participants had the right to withdraw from the study at any time without affecting their medical care or employment rights.

### 2.7 Researcher reflexivity

The interviews were conducted by a graduate nursing student who had received standardized training in qualitative research methods. Throughout the processes of data collection and analysis, the researchers continuously reflected on how their professional nursing backgrounds, research experience, and preconceptions might influence the interview process, data interpretation, and theme development. Regular discussions within the research team, cross-validation of the analytical findings, and continuous self-reflection were undertaken to minimize subjective bias and enhance the credibility of the study.

## 3 Results

Through continuous comparative analysis of interview data from 15 non-shift nurses with a history of night shift work, four themes and 12 subthemes were identified. The four themes were: (1) Night Shift-Related Psychosocial Stressors; (2) Characteristics of Night Shift-Related Intrusive Memories; (3) Contextual Cues and Persistent Experiences Following Night Shift Work; and (4) Coping and Meaning Reconstruction After Night Shift Experiences (Table 3). Based on the identified themes and subthemes, a conceptual model was developed to visually integrate the key findings regarding night shift-related psychosocial stressors, intrusive memory characteristics, contextual cues and persistent experiences following night shift work, and coping and meaning reconstruction. The model was intended as a conceptual representation of participants’ experiences rather than a theoretical explanatory model.

**Table 3:** Themes and subthemes of experiences related to the delayed emergence of night shift-related intrusive memories.

Theme	Subtheme
Night shift-related psychosocial stressors	Discrepancy between expectations and reality
	Perceived organizational inequity
	Professional identity-related distress
Characteristics of night shift-related intrusive memories	Cue-triggered sensory recollection
	Emotional anchoring
	Delayed re-emergence of night shift-related memories
Contextual cues and persistent experiences following night shift work	Environmental cues
	Persistent sleep disturbance after night shift work
	Social reminders and identity-related cues
Coping and meaning reconstruction after night shift experiences	Acceptance-oriented reconstruction
	Boundary-setting reconstruction
	Avoidance-oriented reconstruction

### 3.1 Night shift-related psychosocial stressors

#### 3.1.1 Discrepancy between expectations and reality

Most participants described night shift work as an occupational experience that differed substantially from their initial expectations. Rather than being a personal career choice, undertaking night shifts was often perceived as a requirement resulting from organizational arrangements and job demands. This discrepancy between expectations and reality represented an important psychosocial stressor during their night shift experiences and shaped the emotional significance of subsequent recollections.

Some participants had initially chosen outpatient nursing positions in the hope of avoiding prolonged night shift work. However, their actual work experiences differed considerably from their expectations, which contributed to feelings of frustration and psychological strain.

“I did not take night shifts voluntarily; it was mainly required as part of the rotation training program for new employees and departmental assignments. Most of the time, I had no choice but to accept the arrangement.” (P3).

“One of the main reasons I wanted to work in the outpatient department was that I did not want to work night shifts. However, when I actually started working, I found that the job was not as ideal as I had expected, and the experience was completely different from what I had imagined.” (P11).

#### 3.1.2 Perceived organizational inequity

Some participants perceived the night shift scheduling and remuneration system as unfair. Unequal distribution of night shift duties and a perceived imbalance between workload and financial compensation contributed to feelings of being undervalued and insufficiently recognized. These organizational experiences represented important psychosocial stressors that shaped participants’ emotional responses toward their previous night shift work.

“The distribution of night shifts across some departments is clearly unfair. Some staff members have to work a large number of night shifts, whereas others have comparatively few.” (P5).

“The night shift allowance is so low that it makes you feel your efforts are not valued. A full night shift was paid only a few yuan, which was completely disproportionate to the responsibilities we had.” (P7).

#### 3.1.3 Professional identity-related distress

Some participants reported changes in their perceptions of professional identity after experiencing night shift work. These accounts reflected not only the burden associated with night shift experiences but also broader concerns regarding professional recognition, occupational value, and the perceived imbalance between effort and reward in nursing work. Perceived lack of recognition and a reduced sense of professional value appeared to influence how participants viewed their nursing identity and their willingness to express professional pride.

“In fact, my partner is a manager, but when I have dinner with other people, I rarely mention that I am a nurse, and I do not like talking about my work.” (P7).

“Several members of my family work in healthcare. Over the past few years, I have felt that healthcare professionals are no longer as respected or appreciated as they used to be. Sometimes I even feel a sense of loss about my profession.” (P13).

### 3.2 Characteristics of night shift-related intrusive memories

#### 3.2.1 Cue-triggered sensory recollection

Most participants reported that although they had stopped working night shifts, they still experienced sudden recollections of previous night shift experiences when encountering hospital environments, night-time scenes, or medical equipment. These memories were generally not recalled intentionally but emerged spontaneously when specific sensory cues were encountered, resulting in the re-experiencing of previous situations.

“Sometimes, just walking past the department where I used to work night shifts and seeing the door sign immediately takes me back. It is like a conditioned response.” (P2).

“As soon as I see equipment such as defibrillators or monitors, the feeling of working night shifts comes back all at once. I cannot control it.” (P13).

#### 3.2.2 Emotional anchoring

Most participants reported that memories associated with night shift work were typically accompanied by negative emotions, including anxiety, depression, irritability, and low mood. When these memories were triggered, participants not only recalled their previous night shift experiences but also re-experienced the emotional states associated with those experiences.

“Just thinking about the workload during night shifts makes me feel extremely low. It feels as though my heart skips a beat, and I become completely overwhelmed.” (P6).

“One night, while I was organizing documents, I suddenly remembered those unpleasant experiences of working night shifts. It felt as though there was a huge weight pressing on my chest, making it difficult to breathe.” (P10).

#### 3.2.3 Delayed re-emergence of night shift-related memories

Most participants described that their experiences of night shift work did not completely disappear after they stopped working night shifts. Instead, these experiences could re-emerge months or even years later under specific circumstances, accompanied by emotional responses, reflecting the delayed re-emergence of night shift-related memories.

“I had stopped working night shifts for six or seven years and was busy with my studies. Then one day, I suddenly remembered that unpleasant feeling of working night shifts. It was as though all those experiences came back at once.” (P10).

### 3.3 Contextual cues and persistent experiences following night shift work

#### 3.3.1 Environmental cues

Most participants believed that these intrusive memories did not occur spontaneously but were triggered by environmental cues, including hospital settings, colleagues, and various sensory stimuli associated with night shift work. Exposure to these cues prompted recollections of previous night shift experiences and the re-emergence of the emotions associated with those experiences.

“Now, whenever I see the nurses’ station, a patient room, the emergency department, or even a trolley moving along the hospital corridor, I cannot help remembering my previous experiences of working night shifts.” (P12).

“Whenever I hear a hospital telephone ringing, especially the emergency department ringtone, those feelings come flooding back immediately.” (P14).

#### 3.3.2 Persistent sleep disturbance after night shift work

Some participants described persistent sleep disturbances as one of the lasting effects they associated with previous night shift experiences. Even after discontinuing night shift work, some participants continued to experience difficulty falling asleep, light sleep, and frequent nocturnal awakening. These sleep difficulties reflected the prolonged impact of night shift exposure on their sleep patterns and daily recovery. Unlike environmental cues, these sleep disturbances were not described as direct triggers of intrusive experiences but rather as persistent effects associated with prolonged night shift exposure.

“My sleep has never been good. It has not really improved since I started working night shifts, and it has been extremely difficult to restore a normal sleep-wake schedule.” (P1).

“I have always been a very light sleeper and wake up easily. When I worked night shifts, I was often called during the night to resuscitate patients. Even though I no longer work night shifts, my sleep is still so poor that even the slightest sound wakes me up.” (P13).

#### 3.3.3 Social reminders and identity-related cues

Some participants reported that interactions with former colleagues served as social reminders of their previous night shift experiences. These reminders were associated with complex emotions, including guilt, a sense of loss, and reflections on their previous work experiences.

“Whenever I see my former colleagues still working night shifts, I feel particularly guilty. I cannot help thinking that everyone else has moved on, while they are still there enduring those night shifts.” (P5).

### 3.4 Coping and meaning reconstruction after night shift experiences

#### 3.4.1 Acceptance-oriented reconstruction

Some participants reported that after leaving night shift work, they gradually alleviated the negative emotions associated with their previous night shift experiences through activities such as traveling, exercise, leisure activities, and spending time with family. These approaches were described as ways of readjusting their emotional states and gradually regaining positive feelings toward daily life.

“After going out and spending time with my family, I felt much more relaxed, and those uncomfortable feelings gradually became less intense.” (P8).

“At that time, I was completely exhausted after working night shifts, so I went out to relax and watched a table tennis match. After returning, I felt much lighter, as if I had finally come out of that oppressive state.” (P14).

#### 3.4.2 Boundary-setting reconstruction

Some participants reported that they maintained distance from their previous night shift experiences by refusing to undertake night shift work again. Through this process, they re-established personal boundaries regarding the type of work arrangements they were willing to accept.

“I really do not want to work night shifts again. If I were asked to return to night shifts, I would resist it from the bottom of my heart.” (P6).

“If I were transferred back to the ward to work night shifts now, I definitely could not handle it, and I would never go back.” (P15).

#### 3.4.3 Avoidance-oriented reconstruction

Some participants reported that, in order to reduce the re-emergence of negative experiences associated with night shift work, they actively limited their exposure to night shift-related environments, conversations, or identity-related cues. This avoidance-oriented approach helped them reduce the reactivation of distressing memories in daily life (Figure 1).

**Figure 1:**
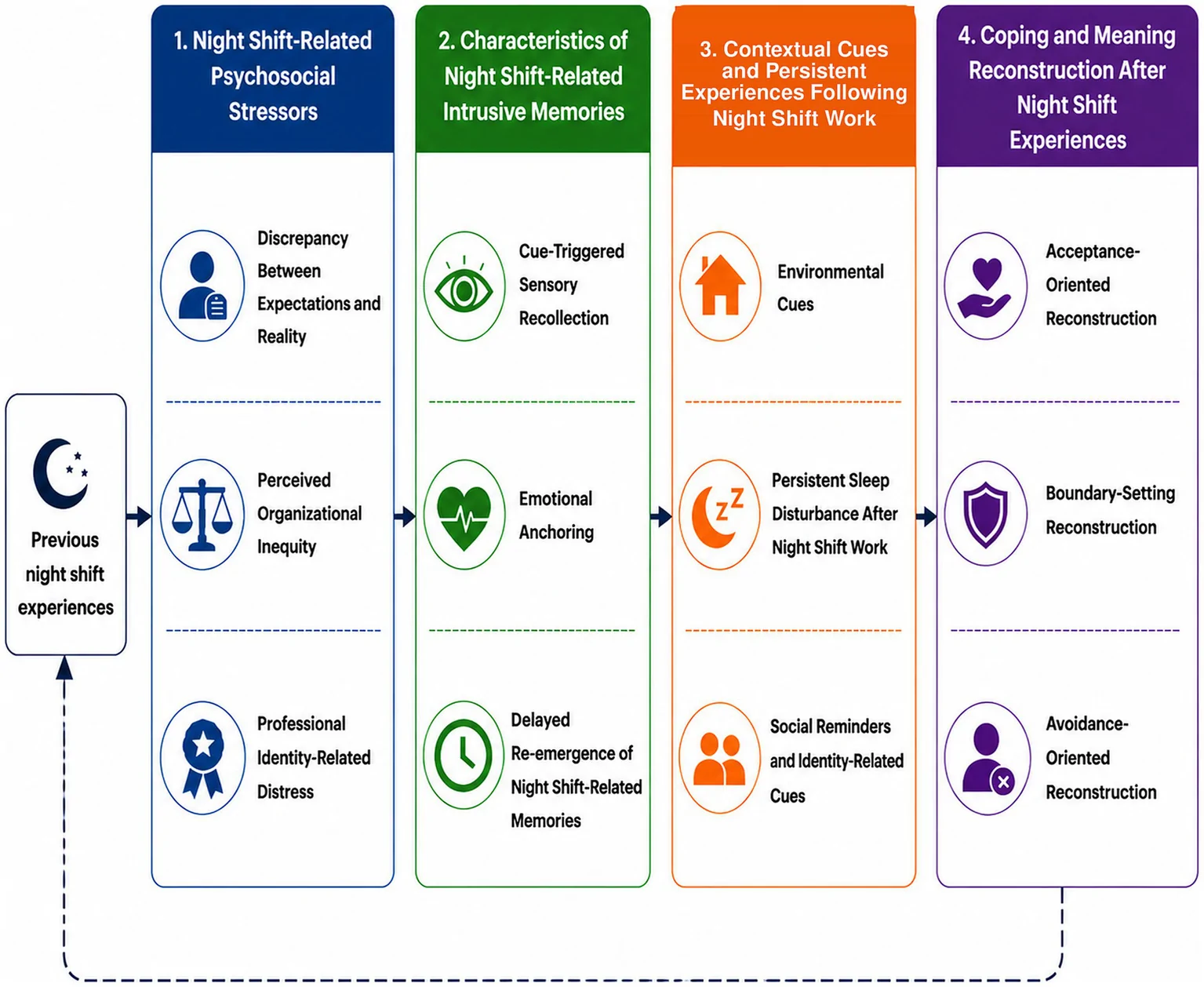
Conceptual representation of the delayed manifestation of night shift-related intrusive memories among non-shift nurses with a history of night shift work. This conceptual representation was derived from an inductive analysis of participants’ subjective experiences and is intended to provide an integrative summary of the identified themes related to the delayed emergence of night shift-related intrusive memories. It does not imply causal relationships among the identified factors, and the interpretations presented in this representation require further validation through future longitudinal and quantitative studies.

“I do not even want to enter the hospital. When I am off duty, I will definitely avoid going to the hospital unless it is absolutely necessary.” (P4).

“Whenever I hear the words ‘night shift’, I feel uncomfortable. I do not want to mention it at all, and I do not want others to bring it up either.” (P9).

## 4 Discussion

### 4.1 The temporal persistence of night shift-related psychological effects and their implications for occupational health

Some non-shift nurses with a history of night shift work continued to experience night shift-related psychological responses under specific circumstances, even months or more than a decade after discontinuing night shift work. These findings suggest that the psychological consequences of night shift work do not simply disappear following a change in work schedule but may instead leave a persistent occupational psychological imprint that remains latent and can be reactivated by specific stimuli, giving rise to conscious psychological experiences (34, 35).

Previous studies have primarily focused on the immediate effects of night shift work, including sleep deprivation, fatigue, depression, and occupational burnout (36), whereas relatively little attention has been paid to the long-term psychological experiences of nurses who have discontinued night shift work (37). By extending the observation period beyond active night shift employment, the present study provides additional evidence regarding the long-term psychological consequences of night shift work and highlights a population that has received limited attention in occupational health management. Unlike previous studies that have regarded discontinuation of night shift work as the beginning of recovery, the present findings indicate that some nurses continue to experience intrusive memories years after leaving night shift work. Hospital environments, medical equipment alarms, and workplace-related cues may trigger recollections of previous night shift experiences, accompanied by intense emotional and physiological responses (13, 38). These experiences are involuntary, context dependent, and transient, characteristics that are consistent with work-related intrusive experiences. These findings suggest that psychological recovery following discontinuation of night shift work is not an automatic process but rather exhibits substantial interindividual variability (39, 40).

Unlike intrusive experiences that develop shortly after acute traumatic events, the delayed intrusive experiences identified in this study appear to result from prolonged and repeated occupational exposure (41, 42). Nursing is characterized by chronic and cumulative occupational stress, which follows distinctive long-term temporal patterns. Therefore, discontinuation of night shift work should not be regarded as the end of occupational exposure. Instead, nurses with a history of night shift work should be incorporated into ongoing occupational health management programs, with long-term follow-up and continuous psychological support provided across different stages of their careers.

### 4.2 Mechanisms of contextual cue activation and implications for the occupational environment

The finding that memories of previous night shift experiences can be reactivated after long periods suggests that environmental cues play an important role in the memory retrieval process (43). Medical equipment, emergency alarms, and hospital environments were frequently identified as triggers, consistent with the concept of context-dependent memory retrieval (44). Experiences of emergency resuscitation, excessive workload, and psychological stress during night shift work may establish stable associations between occupational contexts and emotional responses. These associations may persist long after discontinuation of night shift work and can subsequently be reactivated by similar environmental cues (42).

Some participants also reported persistent sleep disturbances, including light sleep and increased susceptibility to awakening. Although an interaction between sleep disturbances and intrusive experiences is possible, further research is required to clarify the causal relationship between these factors (45). Their co-occurrence suggests a potential association between circadian rhythm disruption and increased sensitivity to memory reactivation; however, the direction of this relationship remains unclear (46).

These intrusive experiences differ from ordinary autobiographical memories and should not be equated with post-traumatic stress disorder (PTSD) (47). Ordinary memories are generally recalled voluntarily, whereas the experiences reported by participants in this study occurred suddenly and involuntarily and were accompanied by pronounced emotional and physiological responses (15). Furthermore, these experiences resulted from prolonged occupational stress rather than catastrophic traumatic events, and most participants remained able to function normally in their work and daily lives. Therefore, it is more appropriate to conceptualize these experiences as part of a continuum of psychological responses associated with long-term occupational exposure within the field of occupational health (48).

### 4.3 The formation process of work-related intrusive experiences from a nursing perspective

This study focused on nursing practice and sought to explain the formation of night shift-related intrusive experiences. The Theory of Unpleasant Symptoms proposes that symptom experiences arise through dynamic interactions among physiological, psychological, and situational factors (49). Intrusive experiences, emotional responses, sleep-related difficulties, and changes in professional identity were closely interrelated, reflecting the multidimensional and dynamic nature of psychological responses after night shift work, which is consistent with the holistic perspective of the theory (50).

Based on the findings of this study, night shift-related intrusive experiences may reflect the cumulative influence of multiple psychosocial hazards embedded in night shift work rather than a single occupational stressor (51). From the perspective of the Job Demands–Resources model, occupational experiences are shaped by the dynamic balance between job demands and job resources (52). In the present study, high workload, emotionally demanding clinical situations, and exposure to distressing events represented important job demands, whereas perceived organizational injustice and limited professional recognition reflected inadequate job resources (53). These findings can also be interpreted through the effort–reward imbalance (ERI) model, which proposes that occupational stress is intensified when sustained effort is not reciprocated by adequate rewards, including remuneration, esteem, and career security (54, 55). Participants’ accounts of demanding night shift responsibilities, low night shift allowances, and limited professional recognition reflected perceived high-effort/low-reward conditions. Although ERI was not formally measured in this qualitative study, this framing helps contextualize how perceived non-reciprocity may contribute to psychological strain and adverse work-related outcomes (56). The cumulative exposure to these psychosocial hazards may have intensified psychological burden over time, contributing to the formation of persistent emotional and memory-related associations. Subsequently, when nurses encountered relevant environmental cues, these previously encoded occupational memories could be reactivated, consistent with the context-dependent memory mechanism (43). When such memories were accompanied by strong negative emotions and psychological distress, they were experienced as persistent intrusive experiences.

It is important to note that the delayed manifestation of intrusive experiences should not be understood as a direct continuation of night shift work, but rather as a dynamic process involving cumulative psychosocial exposures, memory processing, and environmental cues. The formation of mental representations may be influenced by repeated occupational experiences and contextual cue associations, whereas meaning reconstruction may facilitate psychological adaptation and professional identity adjustment (57). The proposed conceptual model was developed through the induction and integration of qualitative findings; however, it remains theoretical and requires further empirical validation. Future research should further clarify the mechanisms through which occupational stress influences psychological outcomes among health professionals (58).

### 4.4 Implications for nursing practice and occupational health management

A comprehensive occupational health management approach should be established to address the psychological risks associated with night shift work (59). At the organizational level, healthcare institutions should prioritize primary prevention strategies, including fair shift allocation, appropriate workload management, adequate compensation, and professional recognition, to reduce psychosocial risks associated with night shift work (60). During periods of night shift work, attention should extend beyond scheduling arrangements to include interventions that reduce occupational stress and role conflict. Following discontinuation of night shift work, regular monitoring of sleep quality, psychological well-being, and occupational adaptation should be considered for nurses with prolonged exposure to night shift work. Appropriate psychological support may help nurses better understand their experiences and facilitate positive meaning reconstruction (57). Improvements in the working environment, organizational support, and institutional care may further enhance nurses’ occupational adaptability and promote workforce stability (61). Occupational health management for night shift workers should therefore extend beyond the period of active night shift work to include ongoing psychological and occupational support. Beyond the Chinese healthcare context, these findings may also have broader implications for occupational health policy and nursing management internationally. Night shift-related psychosocial risks represent common challenges across healthcare systems. Therefore, sustainable occupational health strategies, including prevention-oriented work design, organizational support, and long-term psychological well-being monitoring, may be considered to promote nurses’ occupational adaptation and workforce retention in different settings.

### 4.5 Research limitations and future directions

This study was conducted in a single Grade III Class A hospital in Shandong Province. Therefore, the generalizability of the findings to other regions and healthcare settings requires further investigation. In addition, although participants were encouraged to provide detailed descriptions, the retrospective nature of the interviews may have affected the accuracy of recalling experiences that occurred in the past. Moreover, pre-existing sleep vulnerability could not be completely excluded because participants’ sleep conditions before engaging in night shift work were not objectively assessed. Future studies should consider multicenter longitudinal designs with continuous follow-up of nurses with a history of night shift work after discontinuation of night shifts to more accurately characterize the developmental trajectories of intrusive experiences and identify their influencing factors.

## Data Availability

The data supporting the findings of this study are available from the corresponding author upon reasonable request.
