# Supplementary material for: A qualitative study on the delayed manifestation of night shift-related intrusive memories in non-shift nurses with a history of night shift work
Source: Front Public Health. 2026 Sep 17;14:1938159. doi: 10.3389/fpubh.2026.1938159 (PMC13627312; doi:10.3389/fpubh.2026.1938159)
Supplement: Supplementary file 1 [file Table_1.DOCX]

**Supplementary Table 1. Example of the coding process based on Colaizzi’s seven-step phenomenological analysis**

| Significant statement | Formulated meaning | Subtheme | Theme |
| --- | --- | --- | --- |
| “When I worked in the outpatient department, seeing colleagues from my previous department would make me feel guilty. Seeing resuscitation equipment, such as defibrillators and cardiac monitors, would remind me of my previous night shift experiences.” (P2) | Previous night shift-related environmental and interpersonal cues were experienced as reminders that brought back memories and emotional responses associated with previous night shift experiences. | Environmental Cues | Contextual Factors Associated with Delayed Memory Reactivation |
